# Membrane Blebbing Is Required for Mesenchymal Precursor Migration

**DOI:** 10.1371/journal.pone.0150004

**Published:** 2016-03-01

**Authors:** Beatriz de Lucas, Aurora Bernal, Laura M. Pérez, Nuria San Martín, Beatriz G. Gálvez

**Affiliations:** 1 Centro Nacional de Investigaciones Cardiovasculares (CNIC), Madrid, Spain; 2 Universidad Europea (UE), Madrid, Spain; Beatson Institute for Cancer Research Glasgow, UNITED KINGDOM

## Abstract

Mesenchymal precursors (MPs) present some advantageous features, such as differentiation and migration, which make them promising candidates for cell therapy. A better understanding of MP migration characteristics would aid the development of cell delivery protocols. Traditionally, cell migration is thought to occur only through the formation of lamellipodia. More recently, contractility-driven bleb formation has emerged as an alternative mechanism of motility. Here we report that MPs derived from different tissues present spontaneously dynamic cytoplasmic projections in sub-confluent culture, which appear as a combination of lamellipodia with blebs in the leading edge. Upon initial seeding, however, only bleb structures could be observed. Immunofluorescence revealed the presence of pERM, RhoA and F-actin during the blebbing process. Results from migration assays in the presence of blebbistatin, a myosin II inhibitor, showed that bleb formation correlated with migratory capacity, suggesting a functional role for blebs in migration. Bleb formation might be a useful mechanism to improve cell migration in cellular therapy protocols.

## Introduction

Mesenchymal precursors (MPs) have proven themselves to be useful tools in the area of regenerative medicine since they can repair and/or regenerate a wide variety of damaged tissues [1–3]. Improving the delivery of stem cells that are capable of migrating to sites of damage is a major challenge in cell therapy and is needed to avoid invasive delivery procedures [4,5].

Mesoangioblasts, a subtype of MPs, are vessel-associated mesenchymal stem cells (MSCs) that can be easily obtained from explants of various organs, including heart [6], skeletal muscle [7] and adipose tissue [8]. Heart-derived mesoangioblasts are pre-committed to the cardiac lineage and are endowed with the capacities to migrate [4], engraft into damaged myocardium, differentiate *in vivo* into mature cardiomyocytes and partially ameliorate cardiac function [6]. MP migration has also been reported in MSCs isolated from adipose tissue [5]. Additionally, mesoangioblasts derived from muscle are able to regenerate skeletal muscle in a murine model of Duchenne muscular dystrophy, an X-linked disease caused by an absence of the protein dystrophin [9]. Although the regenerative properties of MSCs are well documented, a vexing question in the field is why the number of cells found in the damaged area rarely correlates with the actual number of injected cells. Accordingly, a better understanding of the homing and migratory properties of MPs is required, and more efficient delivery systems are needed.

Until recently, the lamellipodium has been considered as the be-all and end-all of cell migration. However, many cell types, from amoebae to zebrafish primordial germ cells [10] and mammalian tumour cells [11,12], utilize other forms of structures for motility, such as blebs. Plasma membrane blebs are dynamic cytoskeleton-regulated structures produced by contractions of the actomyosin cortex. The growth of blebs is pressure-driven as a result of strong actomyosin forces that separate a section of the plasma membrane from its underlying cortex, creating a compression of the cytoskeletal network that results in intracellular hydrostatic pressure leading to local bleb expansion [13] that inflates it with cytosol [14]. During expansion, which lasts approximately 30 seconds, the bleb is devoid of actin. Once expansion slows, an actin cortex is reconstituted. The first protein recruited is ezrin, which links the actin cytoskeleton to the membrane and other actin-membrane linker proteins; then actin, actin-bundling proteins and finally myosin motor proteins appear in the blebs. Retraction lasts approximately 2 minutes and is powered by myosin motor proteins through Rho-ROCK-myosin signalling in [15,16]. This process is shared by every cell with blebs, from bovine aortic endothelial cells to HeLa cells [13]. In migrating cells, retraction does not always occur, but instead the cell body moves forward as a result of contraction at the rear [17]. Blebbing is a common physiological feature during cell movement, cytokinesis, cell spreading and apoptosis [15]. Blebbing motility could represent a simpler mode of migration and requires less energy than lamellipodium formation [17,18]. Furthermore, blebs have recently been shown to be involved in stem cell migration *in vivo*, as shown for murine satellite cell [19], or during human MSC transmigration [20,21]. The main factors which have been proposed to control the type of protrusion formed by a cell are actomyosin contractility, actin polymerization, substrate adhesiveness and architecture of the extracellular matrix [22,23]. The proteins RhoA, Rac, and Cdc42 are also involved in this process due to their role in actomyosin contractility and actin polymerization [24].

MSC migration has been extensively studied, however much remains to be learned. In this context, we wished to examine how MPs obtained from adult mice tissues (not only cardiac but also adipose and muscle precursors) migrate *in vitro*. This knowledge of their cellular kinetics and migratory features may improve current stem cell therapies.

## Materials and Methods

### Isolation and expansion of mesenchymal precursors

MPs were isolated from adult mouse tissue explants and characterized as it was described before [25]. Briefly, mice were sacrificed by CO_2_ chamber. Mice were maintained and used in accordance with the National Institutes of Health Animal Care and Use Committee. Protocols were approved by the CNIC Ethics Committee. Adult tissues derived from adipose tissue, cardiac ventricle and skeletal muscle were collected from 4-month-old C57BL6 mice and dissected into 1–2 mm pieces. The tissue fragments were examined under a microscope and those containing small blood vessels were selected. As a new approach, the tissue explants were placed in the centre of 24-plate wells coated with Matrigel (BDbiosciences, Franklin Lakes NJ, USA) around the well perimeter, and were orientated so as to expose blood vessels at the explant border. Explants were cultured in Dulbecco’s modified Eagles’s medium (DMEM, Sigma, St. Louis MO, USA) supplemented with 10% FBS (Sigma,) + Pen/Strep (Lonza, Basel, Switzerland)+L-glutamine (Lonza) + Hepes (Lonza), referred to as complete medium; culture was continued for several days in a humidified 5% CO_2_/95% air atmosphere at 37°C. Afterwards, a population of small, rounded and refractive cells floating above a fibroblast monolayer could be discerned and easily collected with a pipette. Clones were isolated by limited dilution and CFU-F colonies were formed (Three CFU-F colonies from each tissue explant of three different mice were obtained, amplified and characterized). The expansion of these cell populations was performed on gelatin-free culture plates in the same culture conditions. Cells at passage 10 to 20 were used for experiments as indicated. These cells were characterized by flow cytometry. Regardless of their origin cells were CD31 and CD45 negative, while were positive for CD44, Sca1 and CD34 and whereas they showed ckit variable expression. Therefore, these cells are non-haematopietic mesenchymal precursors.

### Inhibition of blebs in cell culture

Blebbistatin (30ng/ml, Sigma) was added to tissue culture medium containing MPs isolated from adipose tissue, heart or muscle. Blebbistatin was added either at the time of seeding (20000 cells) or 24h following seeding of 10000 cells. Images were taken at 1h, 6h and 24h after addition of blebbistatin and the number of cells with blebs were counted in five randomly selected 20x fields using an IX51 Inverted Microscope (Olympus America Inc., Center Valley, PA, USA).

### Concentration of blebbistatin assay

Blebbistatin was added at the moment of cell seeding using the following concentrations: 30 ng/ml, 600 ng/ml and 3000 ng/ml. Representative images of each precursor and condition were taken 24 hours after cell seeding.

### Phase contrast images and videos

Representative images of cultures at short time points were taken with an IX51 Inverted Microscope. Images taken each 5 seconds were stitched together with a one second gap to simulate a video. For long-term imaging, a Nikon ECLIPSE Ti inverted confocal laser scanning microscope (Nikon Instruments, Burgerweeshuispad, Amsterdam, The Netherlands) was used to capture images every 5 minutes for a period of 2 hours. The digital images were then converted into a video file with a 0.4 second gap between images.

### Immunofluorescence

MPs were plated on 0.1% gelatin-coated coverslips and were maintained in culture for 24h before addition of blebbistatin. After incubation, cells were washed with PBS and fixed with 4% paraformaldehyde for 15 min. MPs were then treated with antigen retrieval buffer for 5 min at room temperature (RT) and blocked with 1% donkey serum for 1h. Primary antibodies used were: rabbit anti phospho ezrin/ radixin/ moesin (pERM, 1:100) from Cell Signaling Technology (Danvers, MA, USA), rabbit anti RhoA (1:100) and rabbit anti phospho myosin light chain (pMLC, 1:100) from Abcam (Cambridge, UK). Antibody staining was carried out overnight at 4°C except for pMLC staining, which was carried out for 1h at RT. Immunoreactivity was visualized with conjugated species-specific secondary antibodies (goat Alexa488 conjugated anti-rabbit 1:500) applied for 1h. For actin cytoskeleton staining, cells were incubated with phalloidin-TRITC (Sigma) for 15 min at RT. Coverslips were co-stained with DAPI (300nM) for 30 min and mounted with ProLong Antifade reagent (Invitrogen) on glass slides. Images were observed with a Leica DM2500/TCS SPE confocal microscope (Leica Microsystems).

### Adhesion assay

Matrices (200μl of 1% gelatin, 4mg/ml collagen or 1:1 Matrigel-DMEM) were plated in 24-multiwell plates and incubated for 1h at 37°C. Excess matrix was removed and 10000 cells were seeded and incubated in culture medium for 24h. After this time, cells were counted in five randomly-selected 4x fields using a bright-field microscope (Olympus IX51).

### Wound-healing assay

Confluent cultures of MPs were scratch-wounded with a sterile micropipette tip. After removal of cellular debris, blebbistatin was added and cells were allowed to migrate into the wounded area. Images were captured each hour for 24h with an IX51 inverted microscope using a 4x objective. Wound healing was determined by measuring the wound area using ImageJ software and values obtained were expressed as percentage of wound closure.

### Transwell migration assay

Cell migration was performed with Transwell chambers (Corning Incorporated, Acton, MA, USA) containing 8μm pore size filters. MPs were plated at a density of 2.5 x 10^4^ cells in 80μl medium with or without blebbistatin in the upper chamber of the Transwell and incubated for 24h. The lower chamber consisted of culture medium. After 24h, filters were fixed with 4% glutaraldehyde for 2h and stained overnight with 5% toluidine blue. Cells on the lower side of the filter were counted in five randomly-selected 10x fields using a Nikon 90i bright-field microscope.

### Transwell invasion assay

Invasion assays were performed following a similar protocol to the migration assays but membranes were coated beforehand with 1% gelatin in PBS (3D gel) for 1h at 37°C.

### Data analysis

Statistical analysis was performed using the GraphPad Prism software package (GraphPad, San Diego, CA, USA). Values were expressed as mean±SEM from 3 independent experiments. Comparison between groups was performed with one-way or two-way analysis of variance (ANOVA). The multiple comparisons test used for one-way ANOVA was Bonferroni and for two one-way ANOVA was Turkey. Mann-Whitney test and Student’s t test were also use to compare conditions. The specific analysis use in each graph is specified in the figure caption. Data was considered significantly different when p < 0.05. ns., no significant differences were observed; * P < 0.05; ** P < 0.01; *** P < 0.001.

## Results

### Mesenchymal precursors exhibit spontaneous bleb formation

**Fig 1:**
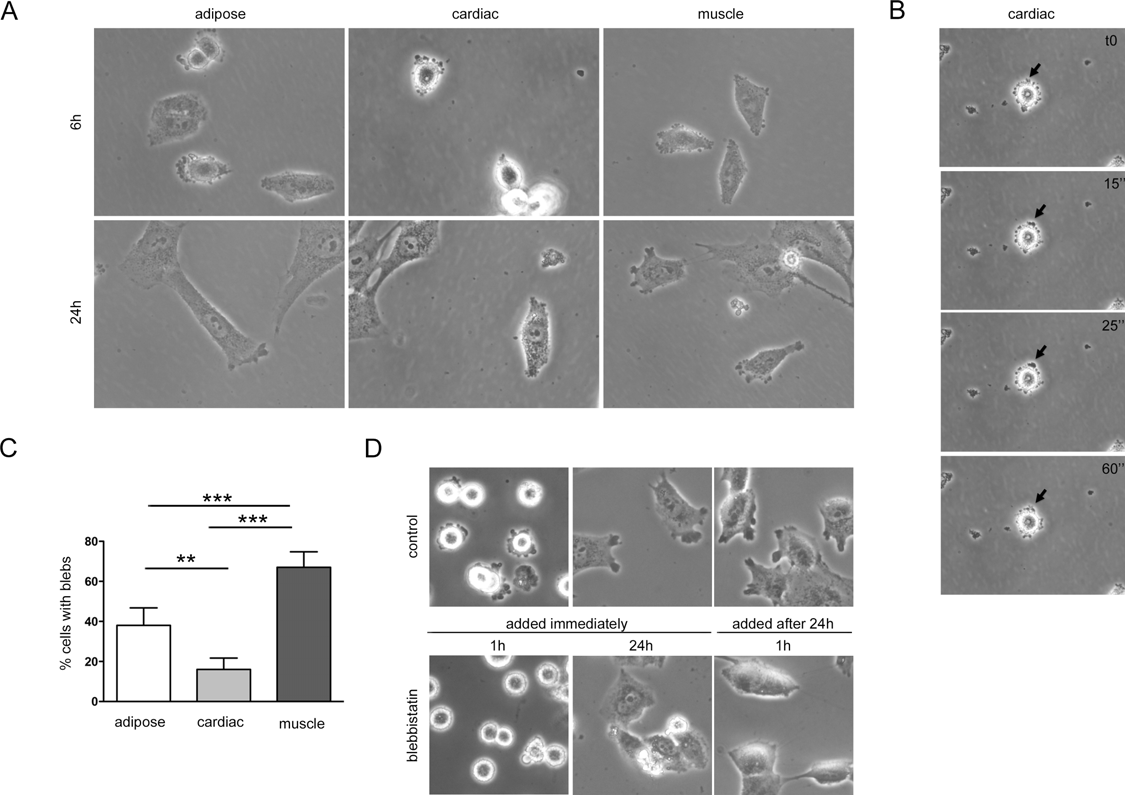
Morphological appearance of mesenchymal precursors in culture. (A) Representative phase contrast images (20x) of MPs at 6 and 24h in culture, where blebbing can be observed. (B) Time-lapse imaging of bleb formation (arrow) in a cardiac precursor, 6h after seeding. Images (20x) show bleb expansion (15” and 25”) and bleb retraction (60”). (C) Quantification of the percentage of cells with blebs in mesenchymal precursors; Statistical analysis were performed using Mann-Whitney test; ** P < 0.01; *** P < 0.001. (D) Blebbistatin-treated muscle precursors (magnification of 20x images), added at the same time of seeding and 24h later respect to control muscle precursors. Images were taken 1h and 24h hours after blebbistatin was added.

Cardiac, adipose and muscle mesenchymal precursors (MPs) were cultured from mouse tissue explants. Almost all isolated MPs exhibited spontaneously dynamic blebs after seeding (Fig 1A). Cells presented a rounded shape and blebs were generalized around the membrane and were the unique type of protrusion detectable at this stage (Fig 1B, S1 Video). At 6h after seeding, cardiac precursors maintained a predominantly rounded shape with generalized blebs, while at this time point, adipose and muscle precursors began to elongate and blebs were polarized and localized at cytoplasmic projections (Fig 1A, S1 Fig). Blebs were observed polarized in the cytoplasmic projections 24h after seeding in all cell types (Fig 1A). Muscle precursors presented the highest percentage of cells with blebs (≈70%) followed by adipose precursors (≈40%) and cardiac precursors (≈20%) (Fig 1C). Blebs located in the cytoplasmic projections appeared as a combination of lamellipodia with blebs in the leading edge and were found in MPs in the non-confluent state. These blebs were less dynamic and smaller compared with blebs in newly-seeded, rounded cells. Live imaging of MPs (S2 and S3 Videos) captured the appearance of blebs at the leading edge of all three precursors, although cardiac precursors appeared to have fewer blebs. S3 Video is especially striking because blebs are relative big and highly dynamics and in a relative short time the cell migrated and retracted changing completely its morphology while the blebs persisted in the leading edge being dynamics.

To inhibit the spontaneous blebbing process, we used blebbistatin, a small molecule inhibitor of nonmuscle myosin II. As expected, treatment with blebbistatin significantly abrogated bleb formation in all MPs tested (Fig 1D, S2 Fig); however, the effects of this inhibitor were dependent on the conditions of the culture. When blebbistatin was added at the moment of cell seeding, cells had fewer blebs and remained rounded for longer periods of time. In contrast, cells were less affected when blebbistatin was added 24h after seeding and only muscle MPs displayed significantly less blebbing (Fig 1D, S2 Fig). Blebbing process has probably an important role for cell adhesion [13] and for that reason cells remained rounded after the treatment with blebbistatin (S2A Fig). Increased concentrations of blebbistatin modified the morphology of cells and affected its viability (S3 Fig).

### Characterization of proteins present in MP blebs

**Fig 2:**
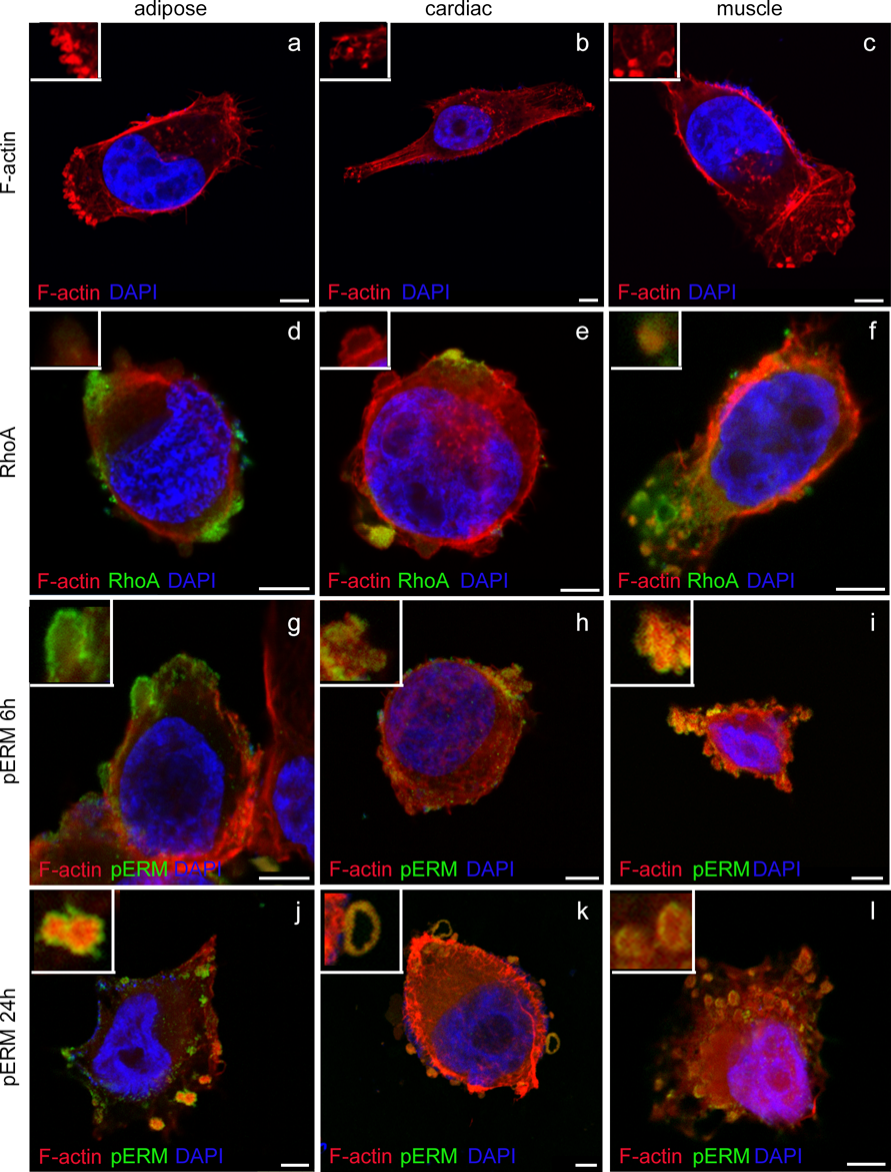
Characterization of bleb structure by immunofluorescence. Confocal images of MPs at 63x using zoom in. Higher magnifications of selected blebs are shown in the upper left corner. All precursors are stained for F-actin (in red) and DAPI (in blue). Cell fixed after 6 hours of seeding the cells were stained for RhoA in green (d-f). Cells were stained for pERM in green at 6 hours (g-i) and 24 (j-l) hours after seeding the cells. Merged of pERM in green, F-actin in red and nuclei in blue at 6h (g-i) and 24h (j-l). Confocal images of MPs at 63x and zoom in. Bar 5 μm.

Immunofluorescence was performed to characterize the protein structure of blebs in MPs. Time course analysis showed that immunoreactive blebs were present at 6h and 24h after seeding and could be observed in the leading edge of MPs in elongated cells (Fig 2A–2C and 2F) and around the plasma membrane in rounded cells (Fig 2D, 2E, 2G and 2H). Blebs could be identified as positively stained for F-actin (Fig 2). Together with F-actin, RhoA and pERM proteins have also been reported as structural constituents of blebs [14,16]. As it has been described in the introduction, each protein plays a role and appears in a different phase of the blebbing process, pERM is the first one to be present (during the bleb expansion) followed up by F-actin assembly and finally RhoA, which is involved in the retraction of the bleb. At 6h after seeding, colocalization of F-actin and RhoA could be observed in a proportion of cells (Fig 2D–2F), whereas F-actin andpERM were observed in others (Fig 2G–2L). In a number of cells, a continuous rim of these proteins could be observed in the plasma membrane (Fig 2C, 2G and 2K). pMLC staining was performed together with F-actin resulting in a light dotted pattern (S4 Fig). Generally migratory cells without blebs present this staining. As it can be appreciated in S1B Fig; b pMLC seems to be present at the neck of blebs (at the leading edge) and there could be involved in the retraction process.

### Bleb dynamics depend on the cellular matrix

**Fig 3:**
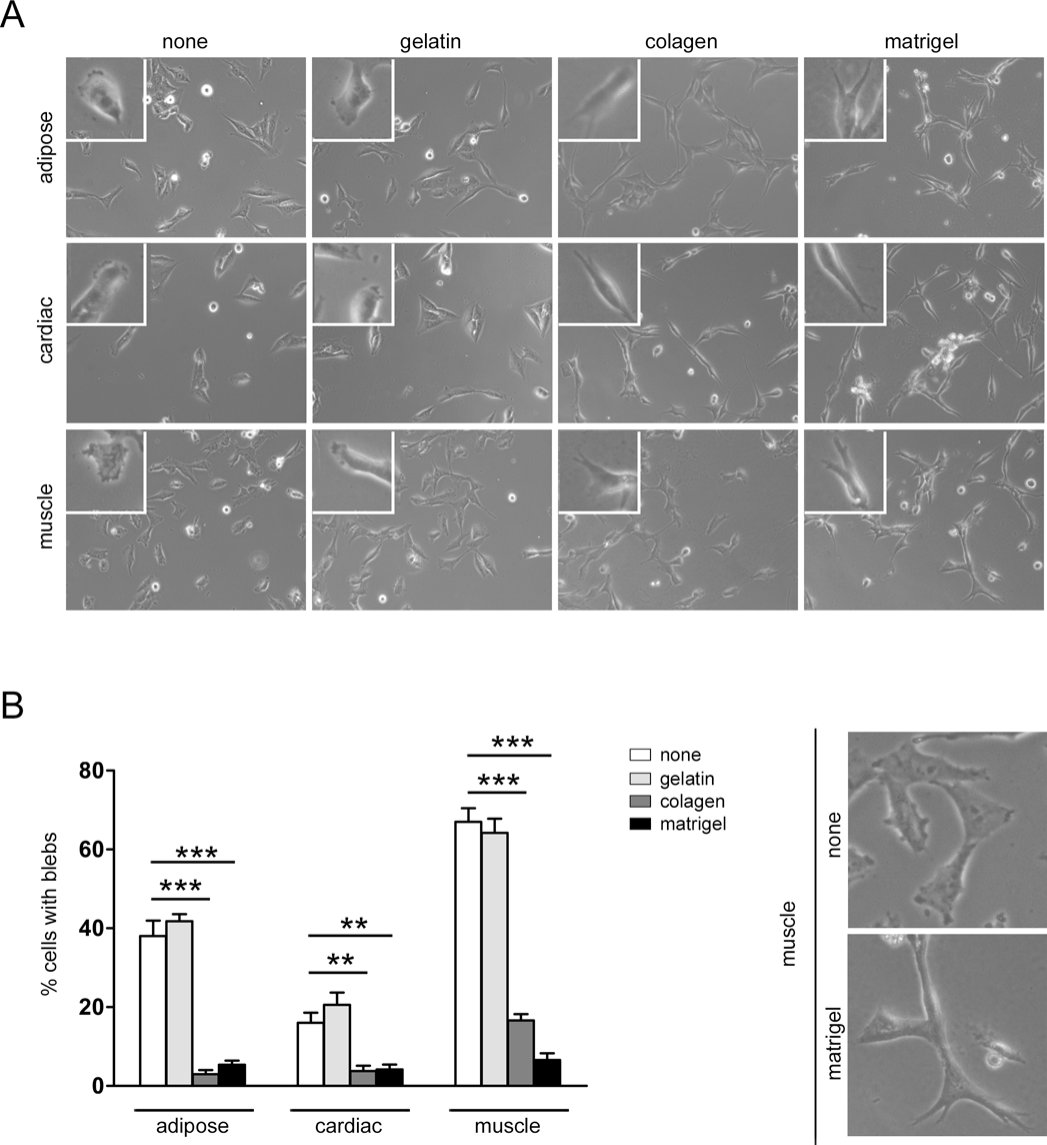
Mesenchymal precursors cultured on different matrices. (A) Representative phase contrast images (10x) of MPs without matrix, on gelatin, collagen and Matrigel. Higher magnifications of selected cells are shown in the upper left corner. (B) Quantification of the percentage of cells with blebs and a comparison of muscle precursor images. Blebbing can be observed in control (no matrix) conditions.; Statistical analysis were performed using One-way ANOVA, Bonferroni; ** P < 0.01; *** P < 0.001.

To examine whether matrix substrata could influence the development of bleb structures in MPs, we performed matrix adhesion assays using a range of basement components. MPs were seeded on different matrices (gelatin, collagen and Matrigel) or uncoated tissue culture plastic, and the morphology of the cells attached to different matrix were evaluated. Changes in cell morphology were observed in MPs attached to collagen and Matrigel compared with those seeded on gelatin or tissue culture plastic (Fig 3A). Accordingly, cardiac and adipose precursors seeded on collagen and Matrigel presented long projections with small bodies (Fig 3A), while muscle precursors exhibited this morphology only on Matrigel. Quantification of cell blebbing showed that seeding on collagen and Matrigel resulted in fewer numbers of blebbing structures in all MPs compared with gelatin basement or no coating (Fig 3B). Furthermore, closer inspection of muscle MPs on gelatin and tissue culture plastic revealed the dynamic nature of the blebbing process. Overall, these results demonstrate that cell morphology and bleb formation are dependent both on the source of MPs and the substratum to which they are attached.

**Fig 4:**
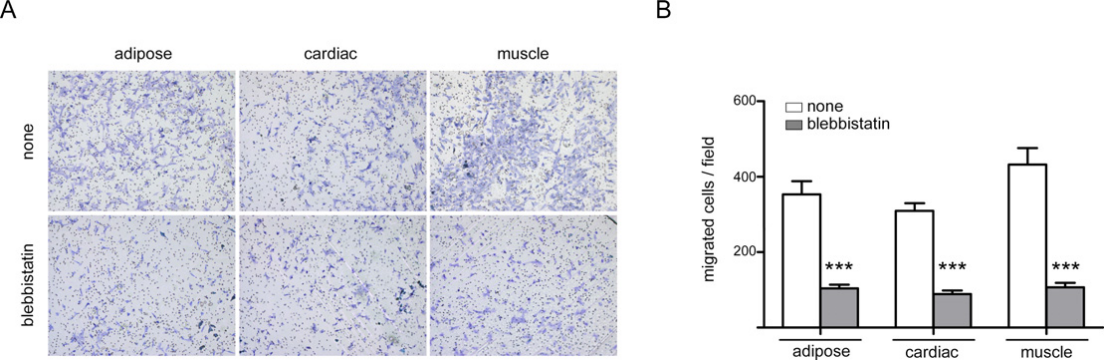
MP Transwell invasion assay. (A) Representative images from five randomly selected 10x fields of MPs with/without blebbistatin are shown. (B) Data are from representative experiment out of three performed and denote mean±SEM of migrated cells/field; Statistical analysis were performed using Mann-Whitney test; *** P < 0.001.

Furthermore we performed an invasion assay to study the role of blebs in 3D matrix migration (Fig 4). Results show significant reduction of invasive capacity when blebbistatin was added to the culture medium (Fig 4A). In control condition the three precursors behaved similarly. To note that the muscle precursors migrated slightly better than the others ones.

### Correlation between migration and blebbing in mesenchymal precursors

**Fig 5:**
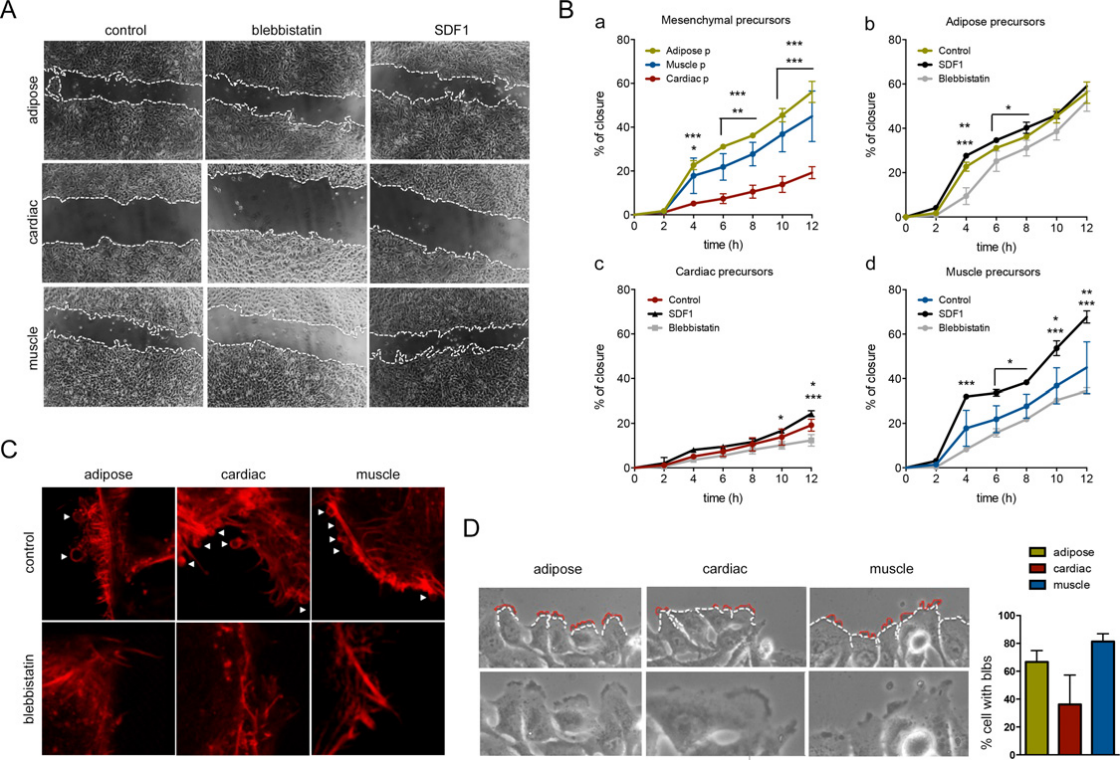
MP wound-healing (migration) assay. (A) Representative 10x images of wounding at 12h are shown. (B) Graphs of wound closure percentage from triplicate samples at each 2 hours over 12h; Statistical analysis were performed using 2 Way-ANOVA and Turkey´s multiple comparisons test to evaluated each condition each hour; * P < 0.05; ** P < 0.01; *** P < 0.001. (a) Comparison of mesenchymal precursors in control conditions (Asterisks above correspond to adipose precursors-cardiac precursors, and asterisk below correspond to muscle precursors-cardiac precursors). (b) Comparison between adipose precursors in control conditions and the same cells with blebbistatin and SDF1 (One row of asterisks refers to SDF1-blebbistatin and in two row of asterisks the above ones are related to control-blebbistatin and the below ones to SDF1-blebbisttatin). (c) Comparison of cardiac precursors in control blebbistatin and SDF1 conditions. (One row of asterisks refers to SDF1-blebbistatin and in two row of asterisks the above ones are related to control-blebbistatin and the below ones to SDF1-blebbisttatin). (d) Comparison of muscle precursors in control blebbistatin and SDF1 conditions. (One row of asterisks refers to SDF1-blebbistatin and in two row of asterisks the above ones are related to control-blebbistatin and the below ones to SDF1-blebbisttatin). (C) Higher magnification of F-actin staining of precursors (63x) in control and blebbistatin conditions. Blebs (marked with white arrows) can be visualized in the membrane of control cells at the leading edge of the cells localized at the front of the wound. (D) Representative phase contrast 20x images of the leading edge of cells that were migrating through the wound are shown. White discontinued lines defined lamellipodia and red lines delimited blebs in the cells. Higher magnification of these images is also shown at the below panel where blebs can be distinguished in black. Quantification of the percentage of cells with blebs at the front of the wound; non statistical differences were found using Mann-Whitney test.

To assess whether MP cell migration involved blebbing, the migratory capacity of MPs was examined with wound-healing assay of confluent monolayers, and the movement of cells into the injured area was monitored over 24h. Compared with cardiac precursor cells, adipose and muscle MPs had a significantly greater capacity for migration (Fig 5A and 5B). At 12h the wound closure was more than 40% in adipose and muscle precursors while cardiac precursors hardly reached to 20%. There are statistical differences between the different precursors starting from 4h (Fig 5B, a). Wound-healing assay were also performed in mesenchymal precursors in the presence of blebbistatin or SDF1 (Fig 5A and 5B; b-d), a chemokine described as an enhancer of cell migration in cardiac precursors [4]. The graphs shown statistical differences in all precursors with the blebbistatin condition at differences hours especially when it was compared with the SDF1 condition. Closer inspection of the leading front of migrating cells revealed that adipose and muscle precursors appeared as individual cells emitting projections, with blebs polarized in the direction of the wound (Fig 5C and 5D). Although bleb projections were also noted in cardiac precursors (Fig 5D), their presence seemed to be lower compared with the other MPs, although any statistical differences were found.

**Fig 6:**
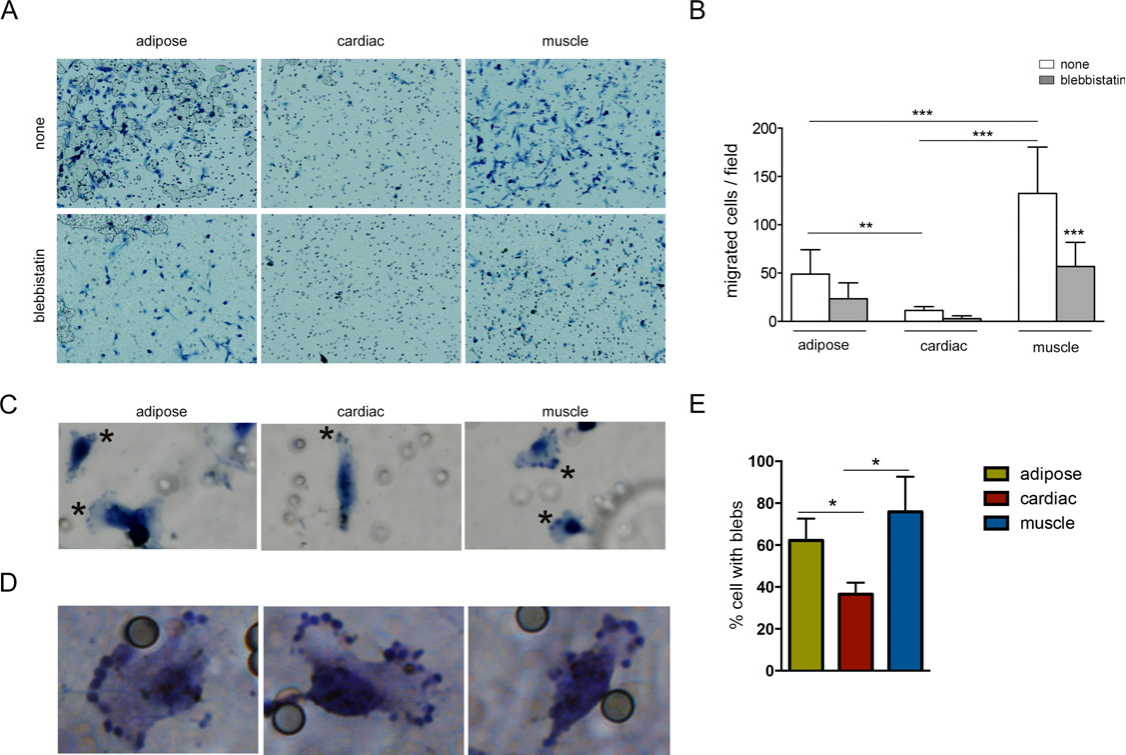
MP Transwell migration assay. (A) Representative images from five randomly selected 10x fields of MPs with/without blebbistatin are shown. (B) Data are from representative experiment out of three performed and denote mean±SEM of migrated cells/field; Statistical analysis were performed using One-way ANOVA, Bonferroni post-test; ** P < 0.01; *** P < 0.001. (C) Zoom of cells located at the lower surface of the Transwell pore, showing fan-shaped lamellipodia containing blebs (marked with asterisks) at the leading edge. (D) Higher magnifications of muscle precursors are shown. (E) Quantification of the percentage of cells with blebs in migrating cells; Statistical analysis were performed using Mann-Whitney test; * P < 0.05.

A more quantitative analysis of migration was performed using Transwell assays, where MPs placed in the upper chamber were allowed to migrate for 24h and migrated cells were scored on the lower surface. In good agreement with the wound-healing assay, results showed that the numbers of migrated skeletal muscle and adipose precursors on the lower surface of Transwell pore were markedly greater than those of cardiac precursors (Fig 6A and 6B). As expected, all MPs demonstrated a reduction in migration (approximately 50%) when blebbistatin was added to the upper chamber, but only this reduction is significant in muscle precursors (Fig 6B). Interestingly, cells migrating to the lower surface of the Transwell pore showed not only large fan-shaped lamellipodia at the leading edge, but also blebs in the membrane of these lamellipodia, particulary in muscle and adipose precursors (Fig 6C–Fig 6E).

Live imaging of MPs (S4–S6 Videos) for 2h following the 24h period of migration captured the appearance of migrating cells containing what appeared to be a combination of lamellipodia and blebs at the leading edge. Collectively, these findings support a correlation between blebs and migratory capacity and demonstrate that blebbing is dynamic, with polarized blebs situated at the leading edge of lamellipodia.

## Discussion

In this study, we demonstrate that MPs from heart, muscle and adipose tissue present blebs spontaneously *in vitro* and these blebs are likely to be related to cell migratory capacity. Long considered a hallmark of apoptosis, blebs have taken an increasingly central stage in the migration capability of cells over the past decade, both *in vitro* and *in vivo* [22]. In MPs, blebs appear just after seeding and are generalized around the plasma membrane of rounded cells. As cells elongate, blebs localize to the leading edge of cytoplasmic projections. These later-formed bleb structures are usually smaller and less dynamic than those formed initially. This change in bleb localization might be explained by their different functions. Blebs generalized around the membrane of a rounded cell could be involved in cell attachment, initiating the spreading process as described for endothelial cells [13], whereas blebs polarized in cytoplasmic projections might be associated with migration, as shown in this study. As expected, immunostaining of bleb protein structures confirmed that MP blebs present p-ERM, RhoA and F-actin, although the composition depends on the bleb dynamic status as reported previously [14,16] indicating that these blebs are active and in continuous development.

Interestingly, polarized blebs appeared to be formed by a combination of lamellipodia and blebs, a coexistence previously described in embryonic and cancer cells [20,22,26]. However, we have observed a combination of these structures not as a simple coexistence. Along this line, Petrie *et al*. have described large blunt, cylindrical protrusions with lateral small blebs termed lobopodia, which have features of both blebs and lamellipodium and are involved in fibroblast migration in specific 3D environments [23,27]. These findings suggest a function for blebs in migration, at least *in vitro*. The clearest evidence of this is the correlation between the presence of blebs and cell migration capacity. Our results show that muscle precursors present the largest number of blebs, followed by adipose and finally cardiac precursors. Cell migration capacity occurs in the same order. Indeed, during migration it is possible to observe the combination of blebs in the leading edge of the lamellipodium in elongated cells. This combination of migration protrusions could be interpreted as an advantage due to the resultant plasticity in cell shape. In fact, it has been suggested that this combination helps cells to optimize their migration in different environments and furthermore bleb migration is independent of proteases [16,17,22,26]. Additionally, blebbistatin-treated cells have a decreased migratory capacity. Although the use of blebbistatin, a myosin II inhibitor, avoids not only blebbing process, but also lamellipodia retraction, we have enough evidences to establish the relationship between the presence of blebs and migration capacity. The clearest one is the correlation between the blebbing activity in MPs and their migration capacity (S5 Fig). Another clue is the localization of blebs in the leading edge of the cells, and as can be clearly appreciated in the videos these blebs are absolutely dynamics during the migration of MPs. Interestingly, blebbistatin appears to have a greater effect on the blebbing process in newly-seeded cells rather than cultured cells, suggesting that blebs are fundamental for cell adhesion; in fact, cells suffer a delay in elongation and remain rounded for longer periods of time.

Previous studies have reported that substrate adhesiveness affects the formation of blebs [22,23]. Usually migration through blebbing occurs in confined and non-adhesive environments [28–32]. We observed different cellular morphologies that were dependent on the substratum. Cells seeded in collagen, presented smaller cellular bodies with very long projections, as has been described [33], whereas cells seeded in Matrigel presented significantly decreased blebbing. This could be due to the fact that these precursors have a greater adhesive capacity to collagen and Matrigel, suggesting that superior cell-matrix adhesion might antagonize bleb formation [20,26,27]. In addition to the preliminary results from invasion assay, blebs could have an important role in cell migration through 3D environments due to the significant decrease in cell migration through the matrix after the use of blebbistatin.

Although not long ago blebbing was mainly associated with apoptosis in the last years, the presence of physiological blebs has been described in a wide variety of different cell types. Many studies in the field are focus on the context where the mesenchymal-ameboid transition occurs in response to environment or internal changes [28]. In this direction, in cancer cells has been described a critical value of compressive traction stress where this transition occurs, at low levels cancer cells show blebs, but at higher stress levels actin stress fibres form resulting in the development of invadopodia-like protusions [34]. In addition, it seems that Rho proteins are overexpressed in tumour and as we had observed these proteins are in close relation with rounded cell morphology and blebs migration. In some cases this correlates with progression of the disease and metastatic potential [30]. But also recently has been found “new” migration structures different from typical blebbing. Many different cell types (immune cell, epithelial cells, mesenchymal cells…) can switch from slow mesenchymal cells to fast amoeboid-like migration under conditions of low adhesion and high confinement. They described two amoeboid-like migration modes [35]. It has been seen in early zebrafish progenitors, an ameboid motility [10] switch into stable-bleb characterized by highly polarized cell morphology with a large spherical protusion front. This is produced with the increase of cortical contractility and have different features from mesenchymal or blebs migration. High spatial confinement combined with low substrate with low substrate adhesion seems to stimulate it [36]. On the other hand a study in mesenchymal stem cells (MSC) demonstrates that stem cells have an inherently weak membrane-cortex adhesion, which increased blebbability thereby regulating cell migration and stiffness in comparison to differentiated cells. After differentiation bleb formation and cell motility is reduced, whereas F-actin organization and the stiffness of the cells is increased [37]. The comparison between freshly isolated articular chondrocytes (in suspension) and the same cells following 9 days in monolayer culture (more fibroblastic phenotype) showed that chondrocytes in monolayer culture has reduced susceptibility to form blebs during aspiration, more cortical actin organization and highest levels ERM and pERM [38–40]. For all that seems to be important to continue investigating about peculiarities of cell migration modes and their plasticity. Blebs are more general that we initially expected, present in many different cell types under special circumstances, especially in stem cells and cancer ones [11,20].

For future approaches it would be necessary to study the molecular mechanisms underlying this combination of migration structures that occurs at the same time and how we can interfere to redirect the migration of the cell to a determinate migration pattern according to our interest.

MPs are promising tools for regenerative medicine; however, efficient delivery and engraftment of cells are major hurdles for successful therapy. Focusing on migration, an increasing number of recent studies point to blebbing migration as an important motility mechanism for *in vivo* migration and movement in 3D environments, and represents a common alternative to lamellipodium-driven migration [15]. In this work, we show that MPs present spontaneous, non-apoptotic and highly dynamic blebs *in vitro*, which appear to be modulated by the same molecular mechanism described for other cell types. Blebs seem to be fundamental for cell attachment, and are needed for migration since blebbistatin significantly abrogates migration, but not all cells present blebs. Blebs could have an important role in cell spreading and migration due to their localization in the leading edge. Hence, blebs may be used by MPs as a mechanism of cell migration in addition to the lamellipodium and this process could be exploited to improve mesenchymal precursor migration and homing for cell therapy.

## Supporting Information

S1 Fig Blebs in MPs. Representative images of different MPs with rounded or elongated shape (magnification of 40x).
(PDF)

S2 Fig Culture of MPs with/without blebbistatin. (A) Representative images (20x) of MPs when blebbistatin was added upon seeding of cells. Graph showing percentages of cells with blebs, data obtained from the images taken at 6h; * P < 0.05. (B) Images of MPs (20x) when blebbistatin was added after 24h of culture. Graph showing percentages of cells with blebs, data obtained from the images taken at 6h; Statistical analysis were performed using t Student test; * P < 0.05.
(PDF)

S3 Fig Concentration of blebbistatin assay. Representative images (20x) of MPs when blebbistatin was added at different concentrations (30ng/ml, 600 ng/ml and 3000 ng/ml) upon seeding of cells. Images were taken 24 hours after cell seeding.
(PDF)

S4 Fig pMLC staining in MPs. Images (63x and magnification) of pMLC and F-actin staining of cells in control and blebbistatin conditions (added after 24h of seeding the cells).
(PDF)

S5 Fig Blebs and their migration capacity. Graph showing the relationship between the presence of blebs in each precursor and their capacity of migration.
(PDF)

S1 Video Cardiac precursors in culture. Cells (40x), 6 hours after seeding without any coating, show large and very dynamic blebs around the plasma membrane. Images were taken every 5 seconds and were stitched together with a one second gap.
(MOV)

S2 Video Adipose precursors in culture. These precursors (40x) show dynamic blebs in the leading edge of elongated cells 24 hours after seeding, without any coating. Images were taken every 5 seconds and were stitched together with a one second gap.
(MOV)

S3 Video Muscle precursors in culture. Muscle precursors (40x) 24 hours after seeding, without any coating present many blebs can be seen for an extended time while the cell was changing greatly in morphology. Images were taken every 5 seconds and were stitched together with a one second gap.
(MOV)

S4 Video Migration of adipose precursors. Blebs can be seen in the leading edge of adipose precursors (20x), 24 hours after seeding, without any coating. Images were taken every 5 minutes during 2 hours and were stitched together with a gap of 0.4 seconds.
(MOV)

S5 Video Migration of cardiac precursors, Cardiac precursors (20x) show blebs 24 hours after seeding, without any coating. Images were taken every 5 minutes during 2 hours and were stitched together with a gap of 0.4 seconds.
(MOV)

S6 Video High migration capacity of muscle precursors. Muscle precursors (20x) have many dynamic blebs in the leading edge. 24 hours after seeding, without any coating. Images were taken every 5 minutes during 2 hours and were stitched together with a gap of 0.4 seconds.
(MOV)
