# Supplementary figures and images for: Membrane Blebbing Is Required for Mesenchymal Precursor Migration
Source: PLoS One. 2016 Mar 1;11(3):e0150004. doi: 10.1371/journal.pone.0150004 (PMC4773234; doi:10.1371/journal.pone.0150004)

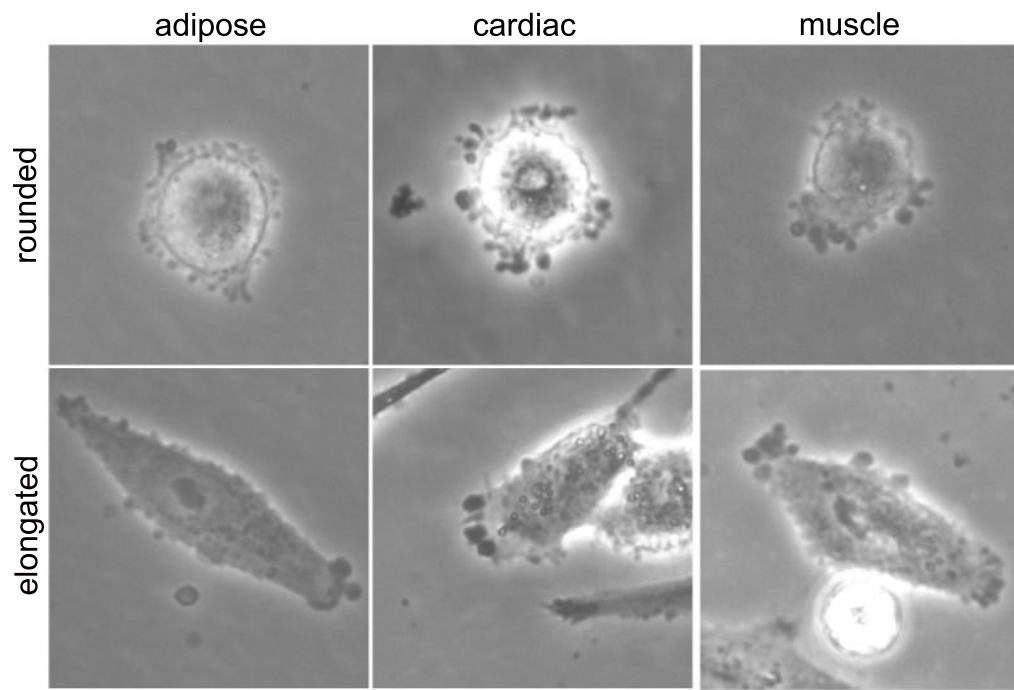

Supplement: S1 Fig — Representative images of different MPs with rounded or elongated shape (magnification of 40x). (PDF) [file pone.0150004.s001.pdf]

A

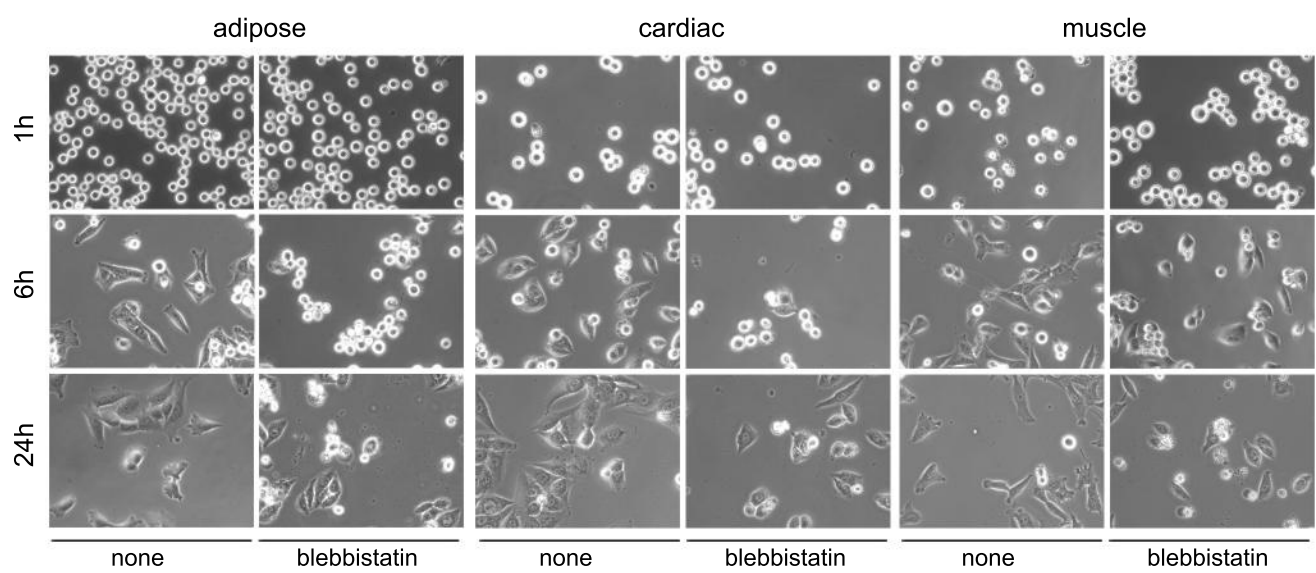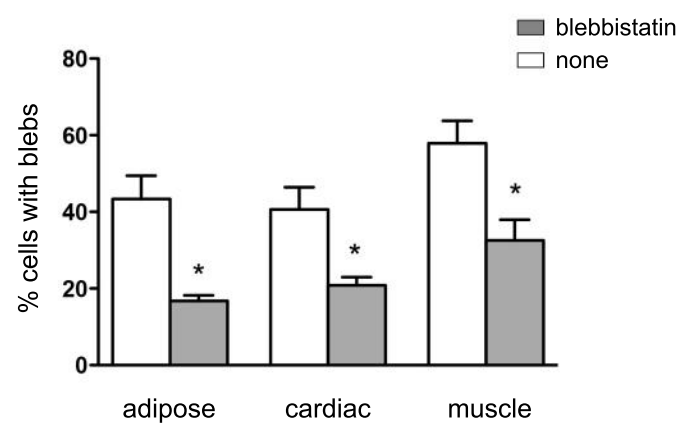

B

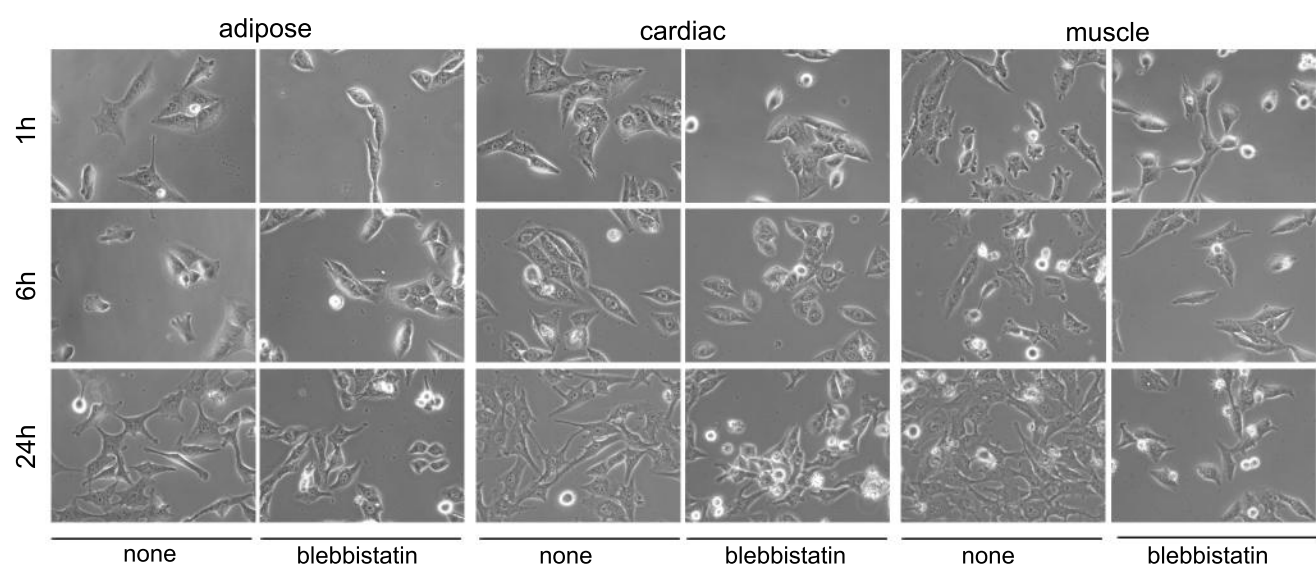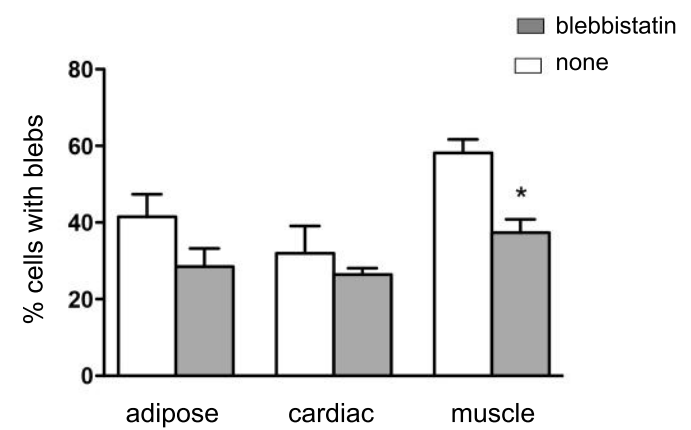

Supplement: S2 Fig — (A) Representative images (20x) of MPs when blebbistatin was added upon seeding of cells. Graph showing percentages of cells with blebs, data obtained from the images taken at 6h; * P < 0.05. (B) Images of MPs (20x) when blebbistatin was added after 24h of culture. Graph showing percentages of cells with blebs, data obtained from the images taken at 6h; Statistical analysis were performed using t Student test; * P < 0.05. (PDF) [file pone.0150004.s002.pdf]

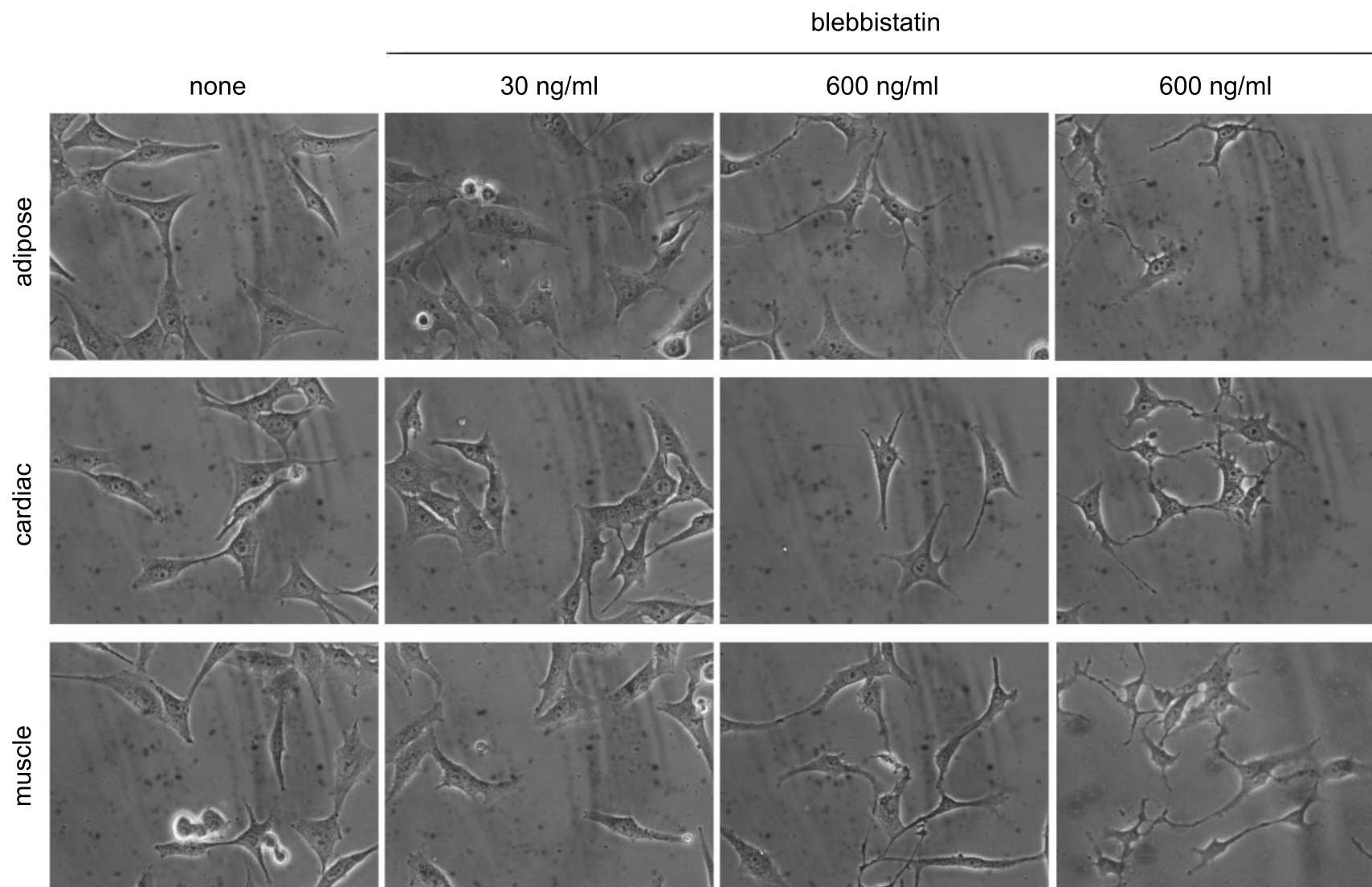

S3 Fig

Supplement: S3 Fig — Representative images (20x) of MPs when blebbistatin was added at different concentrations (30ng/ml, 600 ng/ml and 3000 ng/ml) upon seeding of cells. Images were taken 24 hours after cell seeding. (PDF) [file pone.0150004.s003.pdf]

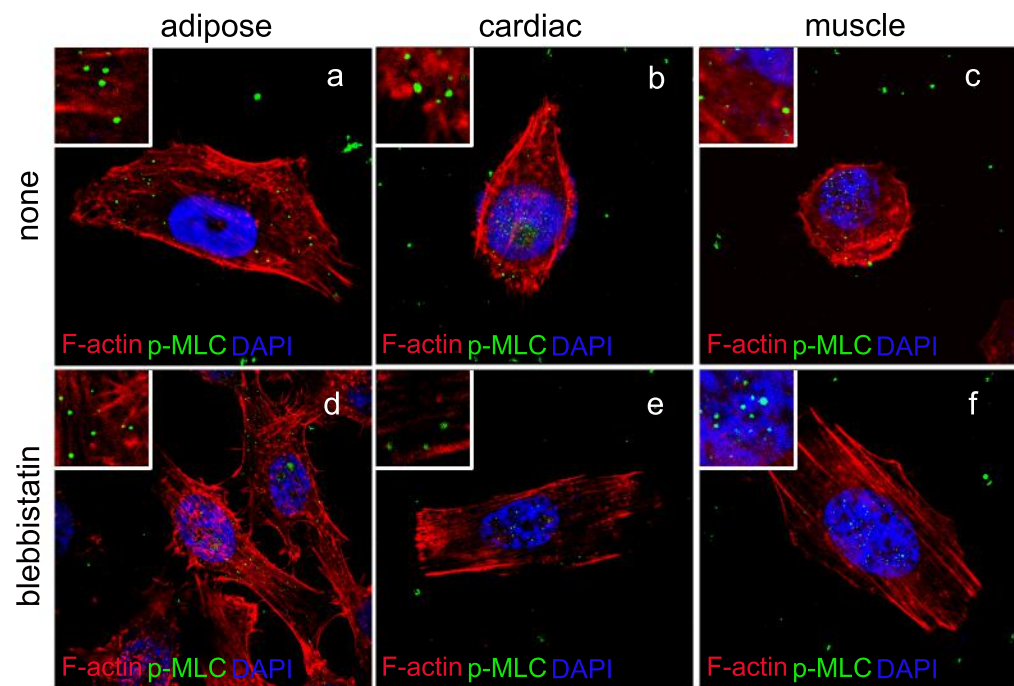

Supplement: S4 Fig — Images (63x and magnification) of pMLC and F-actin staining of cells in control and blebbistatin conditions (added after 24h of seeding the cells). (PDF) [file pone.0150004.s004.pdf]

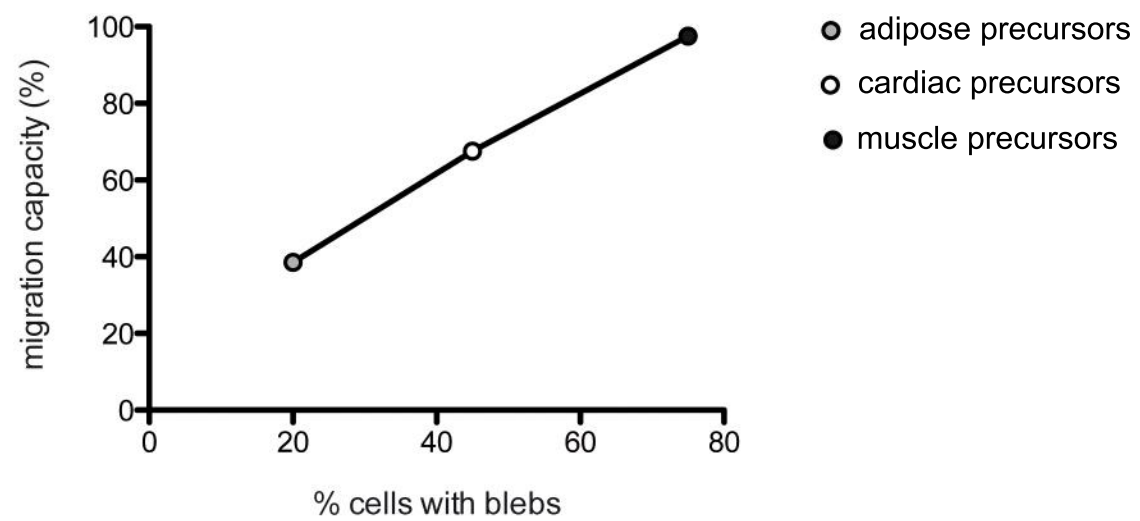

Supplement: S5 Fig — Graph showing the relationship between the presence of blebs in each precursor and their capacity of migration. (PDF) [file pone.0150004.s005.pdf]
